# Towards a feminist global ethics

**DOI:** 10.1080/11287462.2021.2011002

**Published:** 2022-02-10

**Authors:** Rosemarie Tong

**Affiliations:** University of North Carolina, Charlotte, USA

**Keywords:** Global bioethics, feminist global bioethics, rights versus relationships, care versus justice, sameness versus difference

## Abstract

In this article, I explain what makes a global bioethics “feminist” and why I think this development makes a better bioethics. Before defending this assertion explicitly, I engage in some preliminary work. First, I attempt to define global bioethics, showing why the so-called feminist sameness-difference debate [are men and women fundamentally the same or fundamentally different?] is of relevance to this attempt. I then discuss the difference between rights-based feminist approaches to global bioethics and care-based feminist approaches to global bioethics. Next, I agree with a significant number of feminist bioethicists that care is a more fundamental moral value and practice than justice. Finally, I conclude that feminists’ insights about care, even more than rights, can bring us closer to achieving an inclusive, diverse, and fair *feminist* global bioethics.

For many years, I have struggled to articulate what I mean by a feminist global bioethics. Looking back over what I have written about this topic (Tong, 2001; Tong, 2004; Tong, 2005; Tong, 2013; Tong, 2014), I realize the many shortcomings, inconsistencies, and limitations of my thought so far. Nevertheless, I am determined to try, once again, to explain what I mean by a *feminist* global bioethics and why I think it is important for the field of global bioethics in general. I begin with an attempt to define global bioethics before moving on to the so-called feminist sameness-difference debate: are men and women fundamentally the same or fundamentally different? I then discuss the difference between rights-based feminist approaches to global bioethics and care-based feminist approaches to global bioethics. Next, I agree with a significant number of feminist bioethicists that care is a more fundamental moral value and practice than justice. Finally, I conclude that feminists’ insights about care, even more than rights, can bring us closer to achieving an inclusive, diverse, and fair *feminist* global bioethics.

## Introduction: what is global bioethics?

In order to understand what makes a global bioethics feminist, it is necessary to have some understanding not only of global bioethics but also of globalization. According to Diego Gracia, globalization is a relatively new development in the history of humankind, related primarily to advances in information and travel technologies, the creation of profit-driven financial and commercial markets, the modernization of traditional cultures (Gracia, 2014), and, I would add, the emergence of disease pandemics like the recent eruption of COVID-19 in 2020-2022. Similarly, feminists Serena Parekh and Shelley Wilcox observed that “globalization refers to the economic, social, cultural and political practices of integration that result from the expansion of transnational economic production, migration, communications, and technology” (Parekh & Wilcox, 2020). Although some feminists have demonized globalization on the grounds that it systematically exploits women and girls as a source of cheap migrant labor, including sex work (Neuwirth, 2003), global feminist Robin Morgan advised them that because globalization is here to stay, feminists had best repurpose it to serve women’s and girls’ best interests. Morgan saw in globalization an “opportunity to reorder the world in a way that serves humanity - - and particularly the feminist majority - - better” (Morgan, 2003). Diego Gracia supported Morgan’s point, seeing in globalization the possibility to create a just economic market, leveling the playing field between First-World and Third World peoples (Gracia, 2014).

But I am getting ahead of myself. Before there was *global* bioethics, there was simply bioethics, enough of a novelty to cause consternation among some. Contemporaneously, André Hellegers and Van Rennselar Potter began to use the term “bioethics” in the late 1960s and early 1970s. According to Warren T. Reich, for Hellegers, bioethics was a “micro-bioethics,” the application of the standard principles of medical ethics to new developments in medicine and the life sciences. In contrast, for Potter, bioethics was always a “macro-bioethics,” encompassing not only human life but all forms of life including animals, plants, rivers, and so forth (Reich, 1995). By the late 1980s, Potter was confident that he was on the right track, writing that:

“… these two branches must be harmonized and unified in order to be able to arrive at a consensus vision that we can define as global bioethics, highlighting the two meanings of the adjective *global*: an ethical system is global if, on the one hand, it is unified and complete, and, on the other, if it aims at embracing the whole world” (Potter, 1988).

Adding to the attractiveness of Potter’s vision for global bioethics was its focus on the social and environmental problems of people in developing nations as well as those of people in developed nations (ten Have, 2016). Hellegers conceived his bioethics while studying at the Kennedy Institute of Ethics at Georgetown University in Washington D. C. There, he was influenced by the seminal work of Tom Beauchamp and James Childress who co-developed four principles of biomedical ethics: autonomy, non-maleficence, beneficence, and justice (Beauchamp & Childress, 2013). These four principles, sometimes referred to as the “Georgetown Mantra,” were soon exported to Europe, Latin America, Asia, and Africa. To a large degree, Beauchamp and Childress believed their four principles were universal. But according to Henk ten Have, Potter rejected the view that a bioethics

“born, developed and matured in the richest countries of the planet could be exported as a universal model and applied to all countries in the world, with the very realistic risk of establishing a new kind of imperialism - - *bioethical imperialism”* (Pessini, 2018).

Now, at the end of my career, I wish I had paid more attention to Potter’s views at the beginning of my career. I agree with Potter that global bioethics focuses on social and environmental as well as biomedical issues that no one nation can resolve independently. Examples include climate change; natural disasters (hurricanes, tornados, earthquakes, tsunamis); environmental ills (deforestration, pollution); patenting disputes over pharmaceuticals and medical equipment; genetic material controversies, especially those that involve the extraction of DNA from indigenous peoples with rare diseases; the buying and selling of reproductive material (ova, sperm, embryos, wombs) and/or reproductive services (traditional surrogacy or gestational surrogacy); “medical tourism” for many things ranging from inexpensive hip replacements and cosmetic surgery to easily accessible inter-sex or trans-sexual operations; trade in human organs and tissues; and public-health emergencies like the COVID-19 pandemic which disproportionally impacted people in developing nations and/or racial and ethnic minorities in developed nations (McGuire et al., 2020).

I also agree with Potter that global bioethics led to the inauguration of associations like the International Association for Bioethics (IAB), the International Association for Feminist Approaches to Bioethics (FAB), and the International Bioethics Committee (IBC); the publication of many journals on global bioethics; the development of organizations like the World Health Organization (WHO) and the United Nations Educational, Scientific and Cultural Organization (UNESCO); the promulgation of statements like the Universal Declaration on the Human Genome and Human Rights, the International Document on Human Genetic Data, and the Universal Declaration on Bioethics and Human Rights; and the convening of international bioethics conferences from 1992 to 2018 in Amsterdam, Buenos Aires, San Francisco, Tokyo, London, Brasilia, Sydney, Zagreb/Rijeka, Singapore, Rotterdam, Mexico City, Edinburgh, and Bangalore (Pessini, 2018).

But, unlike Potter, I think there is room in global bioethics for a certain type of non-imperialistic, non-colonial “universalism” that is present in some formulations of *feminist* global bioethics. In order to make my case, it is important to state up front that I believe the line between feminist ethics and feminist bioethics is very thin. Thus, I take the liberty of using the expression “feminist ethics/bioethics” from time to time, even when I am referring to thinkers that regard themselves as feminist ethicists with no special interest in feminist bioethics. Moreover, even though I will spend most of this article explaining the difference between rights-based and care-based feminist approaches to global bioethics, I will invoke the work of some global, postcolonial, and transnational feminists as needed (Moghadam, 2005). My goal is both simple and complex: to add a feminist perspective to Potter's global bioethics (Potter, 1971; Potter, 1988; Potter & Potter, 2001), one rooted not so much in women's rights as in the practice of care, a value without which humanity may not make it safely into the twenty-second century.

## Towards a feminist global bioethics: the sameness-difference debate

Like all feminist approaches to ethics/bioethics, a feminist global ethics/bioethics must, as philosopher Alison Jaggar insisted: (1) provide a moral critique of actions, practices, systems, structures, and ideologies that perpetuate the oppression/damaging subordination of women and other groups that suffer comparable or even worse treatment; (for example, people of color, members of LGBTQIA communities, migrants);^1^ (2) consider justifiable ways to resist the economic, social, and cultural causes of the oppression; (3) imagine morally desirable alternatives to the present world as oppressed people experience it - - namely, as sexist, racist, classist, ableist, heterosexist, ethnocentric, nationalist, and/or colonialist; and (4) transform the present world into a future world in which today’s oppressed individuals can thrive (Jaggar 2001).

Initially, most Western^2^ feminists sought to achieve the goals of feminist ethics/ bioethics by stressing women’s *sameness* to men. But the weaknesses of this approach soon appeared. First, by insisting that women can measure up to men, feminists inadvertently conceded that men and men’s traditional domain (the public world) are somehow more valuable than women and women’s traditional domain (the private world) (Chanter, 1998). Second, the reality is that there are many ways in which women’s physical and psychological needs are different from men’s, and deliberately ignoring or downplaying these differences may not be in women’s best interests. One has only to think of the challenges posed by the “pregnant body” - - a body that men do not experience - - to realize this.^3^

Acknowledging that it was probably a mistake to encourage women to deny their differences from men and to unreflectively embrace the traits and values associated with masculinity, some feminists began to present women’s ways of being, thinking, and doing as different or even better than men’s. Carol Gilligan was content to describe men’s and women’s *different* style of moral reasoning (Gilligan, 1982). In contrast, Nel Noddings claimed that women’s ethics were not only different than men’s but also *superior* to men’s (Noddings, 1984).

Unfortunately, the male/female difference was stressed so much that women’s differences from each other were inadequately recognized, as if all women were the same. Gradually realizing that it was incorrect to say that all women have the same interests and values, whether they live in Afghanistan or the United States, for example, most global feminists rejected the idea of “Woman’s” oneness as an essentialist abstraction, implicitly biased toward a certain set of women; namely, white, relatively-affluent, well-educated, healthy, and from a developed nation in the West/Global North (Echols, 1983).

At first, it might seem ethically counterintuitive to reject the idea that all women are the same - - equally worthy of the same respect and consideration. Yet upon careful reflection, it becomes possible to see how the idea of sameness can function oppressively. Feminist philosopher Elizabeth V. Spelman noted, for example, how, in his concern to overcome mid-1950s’ racism in the United States, historian Kenneth Stampp wrote “that innately Negroes are, after all, only white men in black skins, nothing more, nothing less” (Spelman, 1988). Rather than reading Stampp’s words as an enlightened plea for universal brotherhood, Spelman chose to read them as unreflectively racist. Why, she asked, should white men be the standard of “Man” for black men? Why not instead make black men the standard of “Man” for white men?

Spelman’s point and others like it prompted a major change in feminist thought. The idea of “difference” rapidly replaced the idea of “sameness.” In the 1980s and 1990s, many First-World feminists embraced the idea of difference so as not to be viewed as absolutists or colonialists disrespectful of women in the Third World. But there was a serious problem with this well-intentioned move on the part of privileged women. It threatened the kind of feminist thought that requires nations to shape morally just global policies, including ones related to protecting the environment, preserving non-human animal species, fostering human health, and achieving equality between the sexes. Taken to its extreme, the idea of difference, like the idea of sameness, can become an ethical roadblock.

Among others, feminist philosopher Uma Narayan, originally from India, but now living in the United States, noted that many Western feminists tried to abide by two imperatives: (1) “It is important for mainstream Westerners to take an interest in other cultures;” and (2) “It is important that this interest not involve moral criticism of other cultures by mainstream Westerners” (Narayan, 1997). The first of these imperatives, observed Narayan, is rooted in the realization that ignorance about other cultures is “impractical and imprudent in a world where an increasingly global economy reinforces all sorts of complex interdependencies between nations in various parts of the world” (Narayan, 1997). The second of these imperatives, continued Narayan, is based on Westerners recognizing that they are largely responsible for unfavorable representations of Easterners as the “Other:” uncivilized, primitive, barbaric, and/or animalistic. Not wanting to contribute “to a history of negative stereotypes about Third-World communities and practices” (Narayan, 1997), many First-World feminists refused to condemn systems, institutions, and practices in the Third World that they would immediately condemn in the First World. Thus, when Narayan spoke out against sati (a former practice in India whereby a widow threw herself on to her husband’s funeral pyre) or female genital cutting (FGC)/female genital mutilation (FGM), many Western feminists criticized her views as “the analysis of a Westernized feminist [obscuring] the views of the women who actually undergo, or face the prospect of undergoing these practices” (Narayan, 1997). We want, they said, to hear the voices of “authentic insiders” - - *real* Indian women - - not the voices of “inauthentic insiders” - - *faux* Indian women, co-opted by Western influences (Narayan, 1997).

## Rights-Based feminist approaches to global bioethics

Because of the traps of thinking “we are all the same” or thinking “we are all different,” it seems we humans should search for sameness-in-diversity or diversity-in-sameness, an invitation that many rights-based feminist global bioethicists heeded. In the United States in particular, this group of feminist bioethicists claimed that the central function of “rights talk” is to make people of color the equals of white people and women the equals of men, for example. Feminist bioethicist Anne Donchin appealed to the work of feminist legal scholar Patricia Williams to articulate the importance of these “equalizations” and others like them (Donchin, 2004). According to Williams, “[f]or the historically disempowered, the conferring of rights is symbolic of all the denied aspects of their humanity” (Williams, 1991). In other words, said Donchin, we “need to assess the value of rights not only from the privileged position of those who have always had them but also from the position of those to whom they have been denied” (Donchin, 2004). Global ethicist/bioethicist Charlotte Bunch expressed a similar view. She claimed that “the oppression of women in one part of the world is often affected by what happens in another, and . . . no women (Bunch, 1993) is free until the conditions of oppression of women are eliminated everywhere” (Bunch, 1993). Bunch went on to define feminism as “the process” through which women can discuss their commonalities and differences respectfully in an effort to secure the following two long-term goals:

“1. The right of women to freedom of choice, and the power to control our own lives within and outside of the home. Having control over our lives and our bodies is essential to ensure a sense of dignity and autonomy for every woman.

2. The removal of all forms of inequity and oppression through the creation of a more just social and economic order, nationally and internationally. This means the involvement of women in national liberation struggles, in plans for national development, and in local and global struggles for change” (Bunch, 1993).

Bunch’s view reinforced those of global feminist Robin Morgan. All three of Morgan’s most widely-read anthologies - - *Sisterhood Is Powerful* (1970), *Sisterhood Is Global* (Morgan, 1984 & 1990), and *Sisterhood Is Forever* (2003) - - addressed women’s ideas and interests. Of these three books, *Sisterhood Is Global* is of most interest for our discussion. Morgan spent 15 years on this anthology, securing articles from feminists in 80 nations, concluding that so long as women ask each other “*sincere* questions about [their] differences they may discover that they all want a chance to be a self” (Morgan, 1990). The “self” may not be, afterall, a totally Western concept that makes absolutely no sense to women living in the Third World (Nie, 2004). There is within a community, no matter how strong, some space for diversity: for individual thought and action.

Building on Bunch's view, feminist political theorist Susan Moller Okin claimed that feminists must talk about women’s needs “*generically* as well as *specifically*” (Okin, 1994). Conceding that as a group, women do not experience gender inequality to the same extent and degree everywhere, Okin nonetheless insisted that all women do experience oppression in some way or another, for the same reasons, and with the same consequences. Because virtually all societies regard women as the “second sex,” existing to some degree for men’s sexual pleasure, reproductive use, and domestic service, women tend to have less sexual freedom, reproductive choice, good jobs, and free time than men. For this reason, if no other, women’s rights must be recognized internationally, said Okin (Okin, 1998).

Okin’s views and views like hers were voiced beginning in the 1970s at several International Women’s Conferences, including ones in Mexico City (1975), Copenhagen (1980), Nairobi (1985), and Beijing (1995). According to Eschel M. Rhoodie, the first three of these conferences pitted women who were variously labeled as Western, Northern, First World, or from developed nations against women who were variously labeled as Eastern, Southern, Third World, or from developing nations (Rhoodie, 1989). Specifically in Mexico City, some First-World women alleged that some Third-World women were the puppets of their respective governments, instructed to reject women’s rights talk as a capitalist/colonialist ploy to undermine religious practices, cultural norms, and family relationships within their borders (Rhoodie, 1989).

Similarly, in Copenhagen, some women from First-World nations complained that “more heat [was] generated about ‘Zionism,’ ‘racism,’ and ‘Western imperialism’ than about the basic rights of women and their legally deprived status in over 75 of the 118 nations attending” (Rhoodie, 1989). In turn, some women from Third-World nations countered that they had come to Copenhagen wanting to discuss women’s social, economic, and educational concerns, including the plight of Palestinian women and refugees, only to discover that the First-World conference organizers had decided to discuss mostly sexual and reproductive issues, including the wearing of “the veil” (hijab), female genital cutting (FGC), and access to abortion services as priorities without consulting the Third-World women directly concerned. Things got so bad that some conference participants wound up “literally pulling each other’s hair” (Ghodsee, 2010).

Finally, in Nairobi, many First-World women objected that women’s right to birth control didn’t make the list of must-discuss topics in Nairobi, capital of Kenya, “where men’s blind and irresponsible resistance to birth control has produced the highest birthrate in the world, creating catastrophic social and economic problems and condemning women to remain in a centuries old stereotype” (Rhoodie, 1989). In response to this objection, many Third-World women claimed that the Nairobi Conference had been “hijacked” by First-World women who once again wanted to talk about sexual and reproductive issues almost exclusively, implying, said feminist theorist Azizah al Hibri, that the number of infants in the Third World needed to be reduced “in order to preserve the earth’s resources, despite (or is it “because of”) the fact that the First World consumes most of these resources” (Gilliam, 1991).

Mindful of the mistakes made in Mexico City, Copenhagen, and Nairobi, many of the 17,000 delegates from the 189 nations/territories participating in the Beijing Women’s Conference in 1995 came determined to listen to each other’s concerns, commitments, and complaints. Their determination paid off. The delegates to the Conference issued the Beijing Declaration and Platform for Action to secure the human rights of girls and women, and to create “a world in which every woman and girl can exercise her freedoms and choices, and realize her rights, such as to live free from violence, to go to school, to participate in decisions and earn equal pay for equal work” (Miambo-Ngcuka, 2020). Former US Secretary of State Hillary Rodham Clinton added that the Beijing delegates gave her a standing ovation when she proclaimed: “If there is one message that echos forth from this conference, let it be that human rights are women’s rights and women’s rights are human rights, once and for all” (Clinton, 2020).

Unfortunately, as of 2021, none of the 189 nations that signed the Beijing Platform have fully delivered on their promises. Before the COVID-19 pandemic of 2019–2022 hit, the United Nations had planned an October 2020 meeting to speed up the drive for women’s and girls’ rights. But, for obvious reasons, the meeting did not occur (Heilinger, et. al., 2020). Lamenting this situation, *UN Women* Executive Director Phumzile Miambo-Ngcuka stated that: “Research shows the COVID-19 pandemic is exacerbating pre-existing inequalities and threatening to halt or reverse the gains of decades of collective effort with just released new data revealing that the pandemic will push 47 million more women and girls [for a total of 435 million women and girls] under the poverty line” (Miambo-Ngcuka, 2020).

Despite the UN’s promulgation of women’s rights as human rights in international documents like the United Nations Universal Declaration of Human Rights (UDHR) in 1948, the United Nations Convention on the Elimination of All Forms of Discrimination Against Women (CEDAW) in 1979, and the United Nations Declaration on the Elimination of Violence Against Women (DEVAW) in 1993, women in the East, Global South, and developing nations were less enthusiastic about rights talk than their counterparts in the West, Global North, and developed nations. They heard within it a lingering tendency to privilege first-generation rights over second- and third-generation rights.

Czech jurist Karel Vasak is credited with dividing rights into these three generations (Vasak, 1977). He used the three buzz words of the French Revolution - - liberty, equality, and fraternity - - to characterize first-generation, second-generation, and third-generation rights, respectively. Vasak’s distinctions still make sense. First-generation (liberty) rights are primarily civil and political rights, including the right to life, freedom of speech, freedom of religion, the right to property, and the right to vote. Second-generation (equality) rights are primarily economic, social and cultural rights, including the right to be employed fairly and the rights to food, housing, and health care. Third-generation (fraternity or solidarity) rights are the most difficult to itemize, though they seem to include the right to group self-determination, the right to a healthy environment, the right to retain control over natural resources, the rights of ethnic and religious minorities, the right to participation in cultural and religious heritage, and the right to humanitarian assistance (Global, 2019). For the most part, women in developing nations still care more about second- and third-generation rights than first-generation rights. The right to vote, for example, is not nearly as important to them as the right to food, housing, healthcare, and humanitarian assistance.

Other women in the East, Global South, or developing nations rejected rights talk not so much for the reasons just given, but because they thought some of the rights Western global feminists identified as universal were far from being *truly* universal. As they saw it, universal rights are the creation of Western liberalism; that is, they represent only, or primarily, the values and interests that people in nations like the United States favor (McCarthy et. al, 2020). Feminist political theorist Anne Phillips noted that the high value placed on autonomy in statements of universal human rights may be “a central preoccupation of Western cultures” (Phillips, 2002), but not of many Eastern or indigenous cultures “that value the ties of family or community over personal autonomy and mobility” (Phillips, 2002). They do not want to be “liberated” from either the requirements of tradition or the obligations, limitations, and/or benefits that accompany belonging to a community. Global bioethicists Subrata Chattopadhyay and Raymond DeVries reinforced Phillips’ points. They said that the West’s privileging of individual autonomy over societal welfare can be understood

“to contradict the cultural norms and moral values of a major part of the world and to question the foundations of several Eastern religious and spiritual traditions. From the perspective of billions of people in the non-Western world, the idea that [the self has priority over others] is not just absurd, it is dangerous” (Chattopadhyay & DeVries, 2008).

Seeking to take a few steps away from a rights-based approach to achieving social justice, feminist philosopher Martha Nussbaum offered instead a capabilities-based approach to secure the same end. According to Nussbaum, in an original position, people would come together to regulate the structures of society in a fair-minded, co-operative manner (Nussbaum, 2000; Nussbaum, 2002). Assembled in this way, said feminist bioethicist Anne Donchin, people would

“aim at equality of capability rather than equality of resources, since the latter would be more likely to lead to unequal outcomes that could affect them adversely. . . for example, disabled people or those who need more food because they perform hard physical labor might need more resources than others to achieve a comparable quality of life” (Donchin, 2004).

However, in Nussbaum’s estimation, the state does not have an obligation to provide its members with *all* conceivable capabilities “but only those which, if left undeveloped, render a life not human at all, and those which, if left undeveloped, render a human life less than a good life” (Nussbaum, 2002). These fundamental capabilities include “life, bodily health; bodily integrity; senses; imagination and thought; emotions; practical reason; affiliation; other species; play; and control over one’s environment” (Nussbaum, 2002).

Although many feminist activists and theorists in both developing and developed nations applauded Nussbaum’s approach to social justice, some did not. For example, feminist philosopher Daniel Engster claimed that Nussbaum’s list of capabilities does not reflect the needs of *all* women but primarily those of “highly educated, artistically inclined, self-consciously and voluntarily Western women” (Engster, 2005). To this line of criticism, Nussbaum replied that she has no desire to impose a particular conception of a good life on any group of women, or for that matter, any woman other than herself. On the contrary, she insisted her capabilities approach offers each and every woman the resources she needs to decide for herself whether she wants or does not want the norms in her current culture to apply to herself. Indeed, continued Nussbaum, the work of Martha Chen with Indian widows shows that they are highly critical of the cultural norms that determine the quality of their lives, but without the concrete means to do much about it. Offered the resources to change their lives was enough to get some pauperized Indian widows, formerly excluded from mainstream society, to don colorful clothes and even to apply for micro-loans (Nussbaum, 1999). One elderly woman, “widowed” at the age of seven [child brides are not unknown in India], started to dance for the first time in her life, “whirling wildly in the center of the floor” (Nussbaum, 1999). Reacting happily to this scene, Nussbaum commented that given the chance to escape oppressive traditions, there is no good reason for women to “cling” to them (Nussbaum, 1999).

Reacting negatively to Nussbaum’s joy, some of her critics exclaimed “really”???. . . just one week at a widows’ conference to undo years of enculturation and religious formation? Can wearing prettier clothes and applying for a micro-loan to start a small crafts-based business do all of this for a woman nearly overnight? (Seaton, 2020). Further contesting Nussbaum, political theorist and analyst Vivieene Jabri commented:

“. . . Apart from the banality of the certainties expressed [by Nussbaum], there is here a form of ‘epistemic violence’ that astounds. In representing her discourse as a baseline for an international feminism, Nussbaum [engages in] a form of disciplining biopolitics, where the distribution of female bodies and ultimately what can constitute their freedom, as consumers within the global marketplace, where, to use Spivak, ‘to be’ is ‘to be gainfully employed’” (Jabri, 2004).

Apparently Jabri (and Spivak, 1988) were not convinced that enabling Indian women to enter the market place as workers as well as consumers was necessarily liberatory (Molyneux & Razavi, 2002). The women in question might escape the limits of their culture and religion only to be imprisoned in the value schema of advanced-capitalist nations where your worth is dependent on how big your bank account is.

## Care-Based feminist approaches to global bioethics

Bringing us closer to understanding what conditions would make bioethics truly global are, in my estimation, the writings of feminists who refer to themselves as “care-based” thinkers. Although many feminists have articulated the difference between rights/justice-based ethics/bioethics on the one hand and relationships/care-based ethics/bioethics on the other hand, feminist philosopher Eva Feder Kittay has done so in a particularly succinct way. According to Kittay, in rights/justice-based ethics/bioethics

“moral agents are independent, autonomous selves, equal or potentially so; moral relations are ones of rights and relations of equality; the deliberative process is a principled, reason-based calculation that typically occurs in formal contexts; the scope of decisions is such that impartiality and universal applicability are required; the moral aim is to protect against conflict and to adjudicate competing claims; and the signature moral harm is clash between persons” (Kittay, 2006).

In contrast, in relationships/care-based, ethics/bioethics,

“moral agents are relational, dependent selves, unequal in age, capacities and/or powers; moral transactions are ones of responsibilities and relationships of trust; the deliberative process is contextual, narrative, and open to information from the emotions; the scope of decisions is such that partiality is respected and that applicability is context-dependent; and the moral aim is to maintain connections; and the signature harm is broken connections” (Kittay, 2006).

If Kittay’s characterization of care-based ethics/bioethics is correct, people need to develop a certain set of epistemic skills without which they cannot truly care for/care about each other. Feminist philosopher Sara Ruddick claimed that paramount among these epistemic skills are the ones mothers or people who act like mothers typically display: attentiveness, responsiveness, and respect (Ruddick, 1980). Along the same lines, feminist philosopher Virginia Held argued that it is important for people not only to *see* what is wrong with non-maternal/uncaring relationships but also *to do* something about transforming them (Held, 2006). But this is easier said than done. Appeals to self interest will not do. For example, people who reason that all Americans should be provided with universal healthcare insurance because it is cheaper to do this than to pay their maximally-costly emergency room healthcare bills are not engaged in the practice of care. Rather they are engaged in a type of cost–benefit analysis that is, at its core, heartless.

But if self interest is not a proper motive for the practice of care, what about a sense of duty? Philosopher Immanuel Kant’s duty of beneficence is sometimes pointed to as a possible motivator for getting people to engage in Held’s practice of care, albeit “imperfectly” (Kant, 2008).^4^ However, Kant's duty of beneficence is an act of the will, generated by rational powers that resist the interference of the emotions. In contrast, for Held, going through the motions of a caring act without feeling anything in the way of love, affection, compassion, or sympathy is not actually engaging in the practice of care. Convinced that sensitivity to the feelings of others plays an essential role in ethical behavior, Held wondered whether it is at all possible for someone “thoroughly unaware of what others are feeling and thinking, and grossly unable to read the moods and intentions of others. . . to sustain caring relations or [engage] in practices of care” (Held, 2006). Hence it is incumbent on society to raise children’s Emotional Quotient (EQ). All but true sociopaths can learn how to care. Unfortunately, the drive for profit, power, and prestige can deafen one to the cries of others.

Reasoning like Held, feminist political theorist and global bioethicist Fiona Robinson argued that a rights-based ethics/bioethics is too abstract and generalized to take people in the [Global] North

“any closer to mitigating the *actual* suffering of real people caused by continuing poverty. Poverty in the [Global] South is ongoing and part of the everyday lives of those who are, at present, unaware of the way they may be affected by it” (Robinson, 1999).

Robinson conceded that “ … those who would prefer to cling to the familiar language of rights and duties, justice and reciprocity, and the apparent certainty offered to us [people in the Global North] by the kind of ethics which “tells us what to do” and gives us universal standards by which to judge the justice or injustice of all forms of human activity” (Robinson, 1999), may not find the language of care attractive. Indeed, they may find it to be “sentimental, nepotistic, relativistic, paternalistic, and even dangerous” (Robinson, 1999). But the fact that they feel this way is not a decisive reason to bury a care-based ethics/bioethics before it has had a chance to live. Rather, it is an invitation to develop a demanding ethics/bioethics of care that requires people from disparate cultures, religious backgrounds, political orders, and economic systems not only to care-fully listen to each other but also to care-fully work with each other to create and maintain a world in which all people can thrive (Mohanty et al., 2003).

Robinson's thoughts are complimented by those of Maria Mies, a sociologist known for her work in development economics, and Vandana Shiva, a physicist known for her interests in spirituality (Mies & Shiva, 1993). Mies and Shiva stressed that because women, more than men, engage in the work of sustaining daily life, they, more than men, are concerned about the environment. To bear and rear healthy children and provide their families with nourishing food, adequate clothing, and sturdy housing, women need fertile soil, lush plant life, fresh water, clean air, and so forth. In addition, Mies and Shiva pointed out that Western capitalist-patriarchies are characteristically obsessed with the idea of the universal “I”, the overarching “One.” They try to stamp out difference, doggedly cloning themselves, their ideas and their consumable goods wherever they go. In such societies people are alienated from everything: the products of their labor, the beauties of nature, and the companionship of each other. Thus, their capacity for care is gradually squelched and their relationship to nature is tragically distorted (Mies & Shiva, 1993).

Mies described in detail some of the destructive ways in which white men in the Global North try to connect with nature - - the very nature that their lifestyles and patterns of consumption threaten to destroy. First, these men attempt to flee from the confines of their urban offices “into ‘Nature,’ the ‘wilderness,’ the ‘underdeveloped’ nations of the Global South, to areas where the white man has not yet ‘penetrated’” (Mies, 1993). Second, rather than trying to experience the nature in their own backyards, these white men from the Global North seek to experience a more exciting type of nature: nature as “colony, backward, exotic, distant and dangerous, the nature of Asia, Africa, South America” (Mies, 1993). For them, this kind of nature has no intrinsic value; it is simply a commodity to be consumed and then forgotten. Third, these white men from the Global North yearn for yet another kind of nature: “the ‘colored’ bodies of women from the Global South - - wild and dark - - to be penetrated and dominated” (Mies, 1993).

Reinforcing Mies’s analysis of Western men’s unhealthy relationship to nature, Shiva provided an example of 27 northern Indian women’s healthy relationship to it. In order to prevent Western lumberjacks from cutting down their homeland’s indigenous trees, these women chained themselves around them. The Western lumberjacks could not cut down the trees without seriously harming or even killing the women. The women’s protest, known as the Chipko (a Hindi word meaning “to embrace”) Movement, saved thousands of the indigenous trees. Because the women viewed them as integral to their people’s way of life, they were willing to risk just about everything to save them. The Western lumberjacks and, to some extent, many of the men in the village thought the women were acting irrationally. As they saw it, it made more sense to cut down the indigenous trees and replace them with the kind of “high-income generating” eucalyptus trees valued in the West. But even though many of the native men wanted to chop down the indigenous trees as fast as possible so that they could get rich quickly, the native women thought their men were being blinded by Western values (Shiva, 2004). Expressing their care for the indigenous trees poetically, the native women penned some lines that global bioethicist Potter might himself have written.

“A fight for truth has begun

At Sinsyaro Khala

A fight for rights has begun

In Malkot Thono

Sister, it is a fight to protect

Our mountains and forest

They give us life

Embrace the life of the living trees

And streams to your hearts

Resist the digging of mountains

Which kills our forests and streams

A fight for *life* has begun at

Sinsyaro Khala” (Shiva, 2004).

Motivating the Chipko Movement was the belief that because nature is an exhaustible good, and because humankind is a fragile and vulnerable species, all people must develop a subsistence perspective. According to Mies, developing this perspective requires all of the world’s peoples to adhere to “ten commandments” for the protection of the environment, the last three of which are especially important for generating a care-based feminist global bioethics:

“1. Men as well as women should adopt the view of transformative ecofeminism, the subsistence perspective. Specifically, ‘men must focus less on making as much money as possible and focus instead on making their families as loving as possible’ (Mies, 1993).

2. Men as well as women should cultivate traditional feminine virtues (caring, compassion, nurturance) and engage in subsistence production, for ‘only a society based on a subsistence perspective can afford to live in peace with nature, and uphold peace between nations, generations and men and women’ (Mies, 1993).

3. Most important, people should realize that in order for each person to have enough, no person can ‘have it all’” (Mies, 1993).

Kamla Bahsin, an Indian feminist and activist, captured the essence of a subsistence perspective well. She said:

“The standard of living in the North’s affluent societies cannot be generalized. This was already clear to Mahatma Gandhi [who years ago], when asked by a British journalist whether he would like India to have the same standard of living as Britain, replied: “To have its standard of living a tiny country like Britain had to exploit half the globe. How many globes will India need to exploit to have the same standard of living?” From an ecological and feminist perspective, however, even if there were more globes to be exploited, it is not even desirable that this development paradigm and standard of living was generalized, because it has failed to fulfill its promises of happiness, freedom, dignity and peace, even for those who have profited from it” (Bhasin, 1992-1993).

To be sure, a subsistence perspective would require people in the West and the Global North to give up many more resources than people in the East and Global South would have to give up. But the fact that such abstinence would be especially difficult for the powerful and privileged is no justification for the enormous and *uncaring* gap between the rich and the poor in today’s world.

## Conclusion

Back in 2007, there was fear throughout the world that the “avian flu” would be at least as bad as COVID-19, if not worse. During this time, I co-chaired with Leah Devlin, DDS, MPH the North Carolina Institute of Medicine/ Department of Public Health Task Force on Ethics and Pandemic Influenza Planning. She and I co-authored a preface for the Task Force’s report. In it, we appealed to a care-based feminist global bioethics perspective without labeling it as such. The relevant passage reads as follows:

As important as an ethics of justice will be during an influenza pandemic, even more important will be an ethics of care … [For] in the end we human beings are a very vulnerable lot. We are radically dependent on each other for survival, and we need to view ourselves as passengers in a lifeboat in the middle of the ocean with no visible sign of rescue. If there aren’t enough supplies to go around until help arrives, we can do several things: we can ask for volunteers to jump off the boat; we can start drawing straws for who gets pushed off the boat; we can have a majority vote about which lives are most dispensable; or we can look into each other’s eyes and see ourselves - - fearful, hopeful, and in need of compassion - - and then we can start paddling together to get to shore, knowing that although we might not all make it, we didn’t turn on each other in our panic. What we most need to weather a pandemic is an ethics of trust, reciprocity, care, and solidarity. If we have that, we will have the most precious health care resource of all (Devlin & Tong, 2007).

Were Potter alive today, I think he would gladly broaden and deepen his global bioethics with the insights of many feminist bioethicists, but especially those who espouse a care-based perspective. Towards the very end of his life, Potter stated: “I recognize that global bioethics must develop an international bioethics that is politically stimulated and socially engaged: a global bioethics for the twenty-first century requires *care* of people, health and the earth, for all living beings” (Pessini, 2018). To this statement, I say “Amen.”

I do not know what global bioethics will look like in 25 years. I will not be alive then. But I do hope that by the year 2050, say, global bioethics will have incorporated more of the insights of care-based feminists into its core. Like Virginia Held, I think care has a priority over justice. Specifically, she wrote:

“At the level of society, justice now has overwhelming priority, as care is marginalized to private provision or grudging and stingy public support. From the perspective of the ethics of care, this is highly unsatisfactory. Care should at least be on a par with justice, and should perhaps have priority even in the social order, as it certainly has priority in the contexts of family and friends. Consider the case for the priority of care. Care is probably the most fundamental value of all. There can be care without justice: there has been little justice in traditional families but care has been provided. There can be no justice without care, for neither persons nor societies could exist without the enormous amount of care, with its associated values, involved in raising and educating children” (Held, 2004).

If Held has her priorities right, and I think she does, I hope for a future, care-based feminist global bioethics, for unless we human beings learn how to care for each other, beginning with those who have the least among us, we cannot hope to respect each other’s rights, share the world’s resources fairly, protect our common environment, and learn from each other’s diverse traditions, customs, cultural norms, and religious beliefs.

## Notes

1 Feminist philosopher Claudia Card offered a version of feminist global ethics/bioethics similar to that of Jaggar. Card argued that what makes an ethics/bioethics “feminist” is the kind of evils it views as its main object of focus. According to Card, the feminist approach she finds “most helpful takes as its target root evils [intolerable harms] like the oppression and damaging subordination of women and other groups that suffer comparable treatment.” Card continued that “[o]ppression comes in degrees and is not always worse than other wrongs. But, for feminism, oppression is more fundamental than discrimination and often also worse. Salary inequities that wrongly discriminate against female CEOs are unjust but neither evil nor oppressive. Although inequities can contribute to an oppressive pattern, it is good to keep the focus on oppression and treat as a subsidiary concept arbitrary discrimination in distributing the benefits or burdens of social cooperation. In asking, then, whether a group is oppressed, we ask, for example, whether they are locked into a network of practices that stunt their human development systematically, whether they are forced into molds (stereotypes) that deform them, whether they confront an unchosen set of options that whatever they choose they are losers. . . . .” See C. Card (2006). Discrimination against men. Paper delivered at the Eastern Division Meeting of the American Philosophical Association, Washington, D.C., December 30, 2006.

2 Although I realize that essentialistic binaries like West/East, North/South, First World/Third World, white/women of color, and so forth fail to adequately address the true diversity of women, I have, nonetheless, opted to use these terms from time to time. For the most part, I use the First World/Third-World distinction because many global, postcolonial, and transnational feminists use it. No less an authority than postcolonial feminist Chandra Talpade Mohanty defended her use of the First World/Third World distinction in her classic article “Under western eyes: Feminist scholarship and colonial discourses.” Specifically, she said “terms like ‘third world’ or ‘first world’ are very problematical both in suggesting over-simplified similarities between and amongst countries labelled ‘third world’ or ‘first world’ as well as implicitly reinforcing existing economic, cultural and ideological hierarchies which are conjured up in using such terminology. I use the term ‘third world’ with full awareness of its problems only because this is the terminology available to use at the moment. The use of quotation marks is meant to suggest a continuous questioning of the designation ‘third world.’ Even when I do not use quotation marks, I mean to use the term critically.” See C.T. Mohanty (1994). Under western eyes: Feminist scholarship and colonial discourses. In P. Williams, & L. Christman (Eds.), *Colonial Discourses and Postcolonial Theory*. Columbia University Press, 354, footnote 3.

3 In theory, it is possible to get a man pregnant by treating him hormonally and then implanting an embryo in his abdomen or in a donated uterus. See D. Teresi, How to get a man pregnant. *The New York Times Magazine*, November 27, 1994, 54.

4 For Kant, an imperfect duty is one that we do not need to discharge in all circumstances. Rather it is a duty we need to discharge only sometimes and selectively. We cannot be expected to contribute to each and every charity of which we are aware, for example. In contrast, also for Kant, a perfect duty is one we must always discharge. For example, murder and lying are always wrong.
